# Effect of Freeze-Thaw Cycles on the Oxidation of Protein and Fat and Its Relationship with the Formation of Heterocyclic Aromatic Amines and Advanced Glycation End Products in Raw Meat

**DOI:** 10.3390/molecules26051264

**Published:** 2021-02-26

**Authors:** Xingge Wu, Zhigang Zhang, Zhiyong He, Zhaojun Wang, Fang Qin, Maomao Zeng, Jie Chen

**Affiliations:** 1 State Key Laboratory of Food Science and Technology, Jiangnan University, Wuxi 214122, China; 6180111143@stu.jiangnan.edu.cn (X.W.); zyhe@jiangnan.edu.cn (Z.H.); wangzhaojun@jiangnan.edu.cn (Z.W.); qfflast@sina.com (F.Q.); 2 International Joint Laboratory on Food Safety, Jiangnan University, Wuxi 214122, China; 3 State Key Laboratory of Food Safety Technology for Meat Products, Yinxiang Group, Xianmen 361100, China; zzgxmcn@163.com

**Keywords:** pork, freeze–thaw cycles, protein oxidation, lipid oxidation, precursors, heterocyclic aromatic amines, advanced glycation end products

## Abstract

The aim of this research was to investigate the effect of the number of freeze–thaw cycles (0, 1, 3, 5, and 7) on porcine longissimus protein and lipid oxidation, as well as changes in heterocyclic aromatic amines (HAAs) and advanced glycation end products (AGEs) and their precursors. We analyzed the relationship among HAAs, AGEs, oxidation, and precursors and found the following results after seven freeze–thaw cycles. The HAAs, Norharman and Harman, were 20.33% and 16.67% higher, respectively. The AGEs, Nε-carboxyethyllysine (CEL) and Nε-carboxymethyllysine (CML), were 11.81% and 14.02% higher, respectively. Glucose, creatine, and creatinine were reduced by 33.92%, 5.93%, and 1.12%, respectively after seven freeze–thaw cycles. Norharman was significantly correlated with thiobarbituric acid reactive substances (TBARS; r^2^ = 0.910) and glucose (r^2^ = −0.914). Harman was significantly correlated to TBARS (r^2^ = 0.951), carbonyl (r^2^ = 0.990), and glucose (r^2^ = −0.920). CEL was correlated to TBARS (r^2^ = 0.992) and carbonyl (r^2^ = 0.933). These changes suggest that oxidation and the Maillard reaction during freeze–thaw cycles promote HAA and AGE production in raw pork.

## 1. Introduction

Frozen storage is currently the most effective way to preserve meat for long periods of time. Frozen meat is the main form of meat products in circulation, as they are imported and exported in trade among different regions [1]. Compared to room temperature, low temperature conditions can inhibit the growth and reproduction of most microorganisms, reduce enzyme activity and extend the shelf life of meat products. However, in actual production, due to imperfect cold chain technology in transportation, storage, retail, and consumption processes, the temperature fluctuation range is relatively large, so frozen meat products undergo multiple freeze–thaw treatments before they are eaten. These treatments will cause a series of declines in the quality of meat products [2,3].

Many studies have shown that multiple freeze–thaw cycles lead to physiological and biochemical damage in muscle systems, such as loss of nutrients, lipid oxidation, protein denaturation, and hydrolysis [4,5]. Freezing and thawing could cause shrimp protein denaturation, tissue destruction, muscle fiber damage [6], and porcine protein and fat oxidation, especially in pork subjected to five freeze–thaw cycles. Gel electrophoresis patterns of myofibril show that freeze–thaw treatments could cause cross-linking of myofibril [7]. Amino acids and fatty acids in pork change significantly. thiobarbituric acid reactive substances (TBARS) and carbonyl content accumulate, as the number of freeze–thaw cycles increases in the porcine longissimus dorsi [7,8]. The oxidation of lipids can generate aldehydes and free radicals, and the oxidation of proteins can generate active carbonyl compounds. These products can participate in a Maillard reaction to generate heterocyclic aromatic amines (HAAs) [9] and advanced glycation end products (AGEs). It has been reported that high HAA intake is associated with a variety of acute diseases, such as nonalcoholic fatty liver disease and neuronal damage [10,11]. Excessive intake of AGE will result in their accumulation in the human body. Studies have confirmed that AGEs are associated with a variety of chronic diseases, such as kidney failure, diabetes, atherosclerosis, tumors, and Alzheimer’s disease [12,13].

Lipid oxidation and protein oxidation pathways intersect each other and are closely related with Maillard reaction pathways. The Maillard reaction involves a series of complex reactions of carbonyl compounds, which are all initiated by the primary reaction between the reactive carbonyl group and the amino group of amino acids. It is easy to promote the Maillard reaction under high temperature conditions, but the Maillard reaction can proceed slowly under low temperature conditions. There are few publications that report the formation of HAAs and AGEs in raw meat under low temperature. Freeze–thaw cycles cause the oxidation of lipids and proteins, which forms Maillard reaction intermediates. Aldehydes derived from lipid oxidation during freeze–thaw cycles can participate in Maillard reactions, such as acetaldehyde, which is an important intermediate of HAAs, and dicarbonyl compounds, which are important intermediates of AGEs. Hydroxyl radicals derived from lipid oxidation can promote the conversion of fructoselysine (FL) to Nε-carboxymethyllysine (CML) in the glycosylation pathway [14]. HAA formation might be related to the pool of carbonyl compounds that exist in foods, also known as the food carbonylome [15]. In the freeze–thaw state, the change in the physical state of water will affect the secondary or tertiary structure of the protein, which may expose the specific binding site of the protein and the free HAAs, thus facilitating the generation of protein-bound HAAs. Although there are some of reports on the effects of multiple freeze–thaw on pork, most studies are on protein denaturation and fat oxidation.

We analyzed the values of fat oxidation indicators (TBARS) and protein oxidation indicators (carbonyl) of longissimus lumborum at different phases of the freeze–thaw cycles (0, 1, 3, 5, and 7), as well as the generation rules of AGEs and HAAs, and analyzed the relationship between HAAs, AGEs, and their precursors, protein oxidation, fat oxidation during multiple freeze–thaw cycles.

## 2. Results and Discussion

### 2.1. Oxidation of Raw Meat Lipids

Lipid oxidation is an important factor in the deterioration of raw meat quality during freeze storage. Low temperature cannot completely inhibit the activity of endogenous enzymes such as lipase and phospholipase in the muscle. At the same time, chemical reactions can occur in the fraction of unfrozen water, and the freezing of water will increase the contact area between lipids and oxygen. Thus, depending on various factors, the degree of lipid oxidation of raw meat varies in the frozen storage process. Lipid oxidation is exacerbated by the extension of time [16]. Unsaturated fatty acids react with oxygen through free radicals to form peroxides or other primary products. The production of secondary oxidation products (aldehydes, ketones, and esters) will cause raw meat to become rancid.

**Figure 1:**
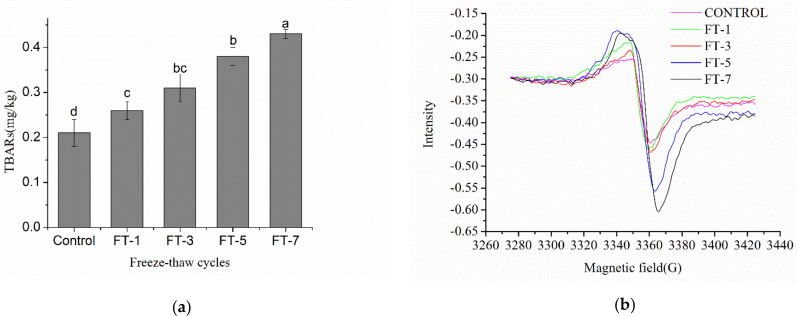
Influence of different freeze–thaw cycles on the thiobarbituric acid reactive substances (TBARS) content of porcine myofibrillar protein (**a**) and the free radicals content of raw meat (**b**). Freeze–thaw(FT)-1, FT-3, FT-5, and FT-7 represent freeze-thawed 1, 3, 5, and 7 times, respectively. Means (*n* = 3) across all samples without a common letter differ significantly (*P* < 0.05).

The higher the TBARS content, the stronger the lipid oxidation will be. Figure 1a shows the change in the TBARS content of raw meat throughout the freeze–thaw cycles. The TBARS content of raw meat increased with the number of freeze–thaw cycles (*P* < 0.05), rose from 0.21 ± 0.03 to 0.43 ± 0.01 mg/g after seven freeze–thaw cycles, which indicated that the fat of raw meat continued to oxidize throughout the addition freeze–thaw cycles, and the content of malondialdehyde also increased. The TBARS content of raw meat increased after one freeze–thaw cycle and reached the highest concentration after seven freeze–thaw cycles. The primary oxidation of lipid was more likely to increases post-thawing and to promote the formation of secondary lipid oxidation, which is consistent with the findings of Ali et al. [2]. Pan et al. [17] reported the similar conclusion that there was a distinct positive correlation between freeze–thaw cycles with lipid oxidation in pork patty. When the raw meat underwent the freeze–thaw cycles, ice crystals induced damage to muscle tissue microstructure, which may lead to the release of pro-oxidant factors (oxidized lipids, free radicals, and heme pigments), enzyme (lipase, nuclease, and protease), and oxidation enzyme precursors inside cells, thus accelerating the lipid oxidation during freeze–thaw cycles [18,19].

The formation of free radicals can significantly promote lipid oxidation and plays a vital role in accelerating the oxidation process. Free radicals are also associated with the formation of HAAs and AGEs [20]. Figure 1b shows the change in the free radical content of muscle samples during the frozen storage process. The difference in signal intensity indicated the free radical content of raw meat. The free radical content in pork increased after three freeze–thaw cycles and rose to the highest level after seven freeze–thaw cycles (*P* < 0.05). Yu et al. [21] found that the content of free radicals keeps increasing in frozen raw meat during storage, which is consistent with our research. This formation of free radicals may be derived from the oxidation of lipids and proteins, and oxygen can promote the generation of free radicals in meat products during storage [22]. Free radicals also participated in and promoted the oxidation of lipids and proteins.

The changes in fatty acid composition during the freeze–thaw cycles of raw meat are shown in Table 1. With increase in the freeze–thaw cycles, the fatty acid content of the muscle sample changed significantly (*P* < 0.05). The contents of saturated, monounsaturated, and polyunsaturated fatty acids decreased with the increase freeze–thaw cycles. Most fatty acids reduced after one freeze–thaw cycle and kept stable after five freeze–thaw cycles. Hernández et al. [23] found a decrease in polyunsaturated fatty acids (PUFA) after 6 months of frozen storage in pork. Igene et al. [24] came to the same conclusion in beef during frozen storage. The change in fatty acids during frozen storage involves the oxidation of fatty acids and the hydrolysis of phospholipids and triglycerides. The early stage mainly involves lipid oxidation, and the later stage mainly involves the hydrolysis of lipids. This general decrease in PUFA could be accounted for the enzymic hydrolysis concentration during frozen storage, because lipolytic enzymes remain active at freezing temperatures. At the same time, the freeze–thaw process facilitates the release of enzymes due to the growth of ice crystals and cell rupture.

### 2.2. Oxidation of Protein in Raw Meat

The freeze–thaw treatment will cause a series of physical and chemical changes in protein, such as changes in the structure of muscle fibers (larger gaps, shrinkage, or breakage), oxidative denaturation and degradation of proteins, and amino acid changes. The carbonyl compounds generated by protein oxidation can participate in the Maillard reaction and promote the production of related products. The carbonyl content was used to evaluate the degree of muscle protein oxidation.

**Figure 2:**
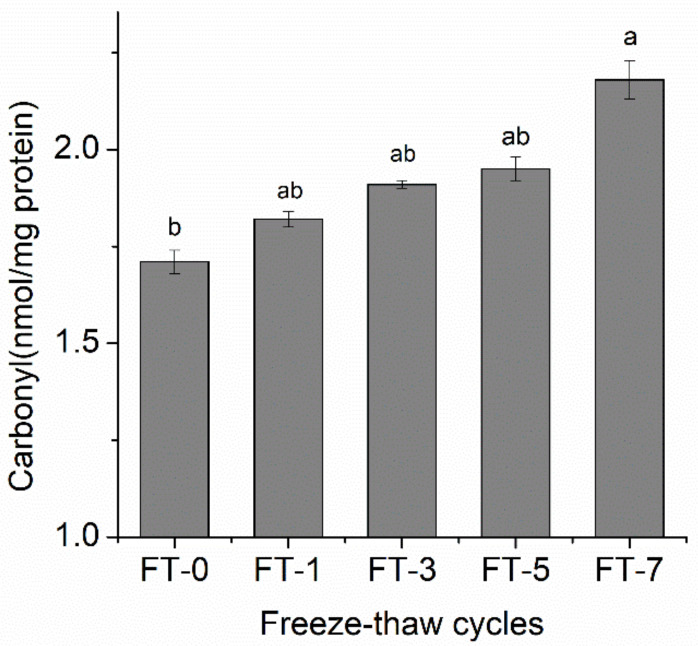
Influence of different freeze–thaw cycles on the carbonyl content of porcine myofibrillar protein in raw meat. FT-1, FT-3, FT-5, and FT-7 represent freeze-thawed 1, 3, 5, and 7 times respectively. Means (*n* = 3) across all samples without a common letter differ significantly (*P* < 0.05).

Protein carbonylation occurs during slaughter, processing, and refrigeration [25]. Figure 2 shows variations in the carbonyl content of the muscle sampled during frozen storage. The carbonyl content increased, as the number of freeze–thaw cycles increased (*P* < 0.05). In the blank muscle sample, it was lowest (1.71 ± 0.03 nmol/mg protein), and after seven freeze–thaw cycles, it reached highest amount (2.18 ± 0.05 nmol/mg protein). This indicated that the protein oxidation in muscle increased as the number freeze–thaw cycle increased. Our results are in line with those of Pan et al. [17]. During frozen storage, protein oxidation in muscle can be caused by a variety of factors, such as hydroxyl radicals [26], myoglobin free radicals, or lipid secondary oxidation products (malonaldehyde) [27].

Table 2 shows the changes in the free amino acid content of pork during frozen storage, and a total of 14 free amino acids in the muscle sample were detected. As the number of freeze–thaw cycle increased, some of the free amino acids also increased, such as Gly, His, Arg, Ala, Met, Leu, Ile, Phe, and Lys (*P* < 0.05), mainly due to the formation of ice crystals, which destroyed the integrity of muscle tissue and caused muscle fiber breakage and protein oxidation and degradation. At the same time, a series of hydrolases involved in protein degradation and hydrolysis after thawing also caused an increase in the content of free amino acids. Kim et al. [28] analyzed the components of the exudate during the thawing of frozen meat and found that it mainly contained water-soluble sarcoplasmic proteins, nucleotides, amino acids, peptides, proteins, and a large number of soluble enzymes, which also confirmed our conclusions. Amino acids are related to the nutritional value and flavor quality of meat and meat products and are also important precursors of some reaction products, such as HAAs and AGEs. A large number of experiments of the model system have shown that a variety of amino acids can participate in the formation of HAAs [29,30,31]. Both free amino acids and protein-bound amino acids can participate in the production of HAAs and AGEs; however, free amino acids react quickly with the precursors of related HAAs and AGEs. Therefore, only free amino acids were measured in this paper, and their variation can explain the generation of HAAs and AGEs to a certain extent.

### 2.3. Glucose, Creatine, and Creatinine

Table 3 shows that the glucose content gradually decreased (*P* < 0.05) with the number of freeze–thaw cycle increase. It dropped significantly during the third freeze–thaw cycle, decreased from 29.19 ± 1.15 to 18.32 ± 3.21 µg/g and then stabilized. This may due to cell rupture during freeze–thaw cycles and glucose lost with the juice. At the same time, glucose is an important precursor or substrate of many products that can directly promote the formation of HAAs and AGEs and can also cause the accumulation of highly active dicarbonyl compounds [32]. Glucose and dehydrating amino acids cyclize to form pyrazine and pyrrole, and they participate in the generation of HAAs. If carbohydrate is added to the actual system, the amount of HAAs in the later stage will be affected, which is promoted at a low concentration but inhibited at a high concentration [33]. After the slaughter of livestock, glucose, ribose, and other components in the muscle participate in the Maillard reaction. The dicarbonyl compound can induce oxidative deamination of amino residues in proteins to form α-aminoadipic semialdehydes (AAS) and Γ-glutamic semialdehydes (GG-glutamic semialdehydes, GGS) [34,35].

Creatine and creatinine are the precursors of various HAAs. Table 3 shows the content of creatine and creatinine changed significantly after a freeze–thaw treatment (*P* < 0.05), with reductions of 16.12% and 4.31%, respectively, which may be due to the reason that creatine and creatinine are water-soluble and part of them is lost during the thawing process. Adding creatine and creatinine to the system will have a certain effect on the production of HAAs [36]. Zeng et al. [37] studied the effects of different precursors on formation of HAAs and found that creatine and creatinine have a certain effect on the formation of HAAs. Specifically, Lee et al. [38] showed that creatine and creatinine promoted the production of HAAs when heating the gravy.

### 2.4. HAAs and AGEs

As shown in Table 4, for HAAs, different levels of Norharman and Harman were detected in the muscle samples, whereas other types of HAAs were not detected. The contents of Norharman and Harman increased significantly with the increase of freeze–thaw cycles (*P* < 0.05), rose from 116.03 ± 8.24 to 139.62 ± 6.61 ng/g and from 88.12 ± 8.90 to 102.81 ± 14.59 ng/g, respectively, after 7 freeze–thaw cycles. Changes in HAA content in raw meat may be related to changes in amino acids, glucose, creatine, and creatinine during frozen storage. HAAs can be generated by Maillard reaction [39]. During the freeze–thaw process, the moisture in raw meat repeatedly underwent the freezing and thawing, which accelerated the movement of water-soluble precursors of mutagenic compounds (reducing sugar, amino acids, and creatine), which may promote the formation of HAAs. Persson et al. [40] found that adding carbohydrates such as potato fiber, wheat bran, potato starch, methyl cellulose, and guar gum to beef patties decreases the movement of HAAs precursors and reduces the formation of HAAs. Although Norharman and Harman have no exocyclic amino groups and are not teratogenic, they will significantly promote the mutagenicity of other HAAs under long-term exposure [41]. Studies have pointed out that β-carboline HAAs, such as Norharman and Harman, can form as a result of a reaction of glucose and amino acids below 100 °C [38].

AGEs are generated in foods as advanced reaction products derived from a Maillard reaction. CML and CEL are usually recognized as typical AGEs in the food industry, because of their stability. Table 4 shows the changes in the CEL and CML contents of raw meat during freeze–thaw cycles. Different levels of CML and CEL were detected in raw meat. As the number of freeze–thaw cycles increased, CML in pork gradually accumulated (*P* < 0.05), up to 48% higher than in the blank muscle sample, and CEL was 8% more than in the blank muscle sample. Raw meat inevitably underwent protein and lipid oxidation. These oxidative changes (such as the breaking of peptide bonds and the exposure of reactive amino acid residues, dicarbonyl compounds, and free radicals produced by lipid oxidation) may promote the production of CML and CEL through the Maillard reaction pathway. Zhu et al. [42] studied chicken breast and found that protein and lipid oxidation could promote AGEs formation. At the same time, endogenous AGEs are synthesized in animals, and reducing sugars in meat react spontaneously with the amino acid residues of proteins, lipids, and nucleic acids to form AGEs at a later stage [43].

### 2.5. Association of HHA and AGE with Oxidation and Precursors

Important precursors of HAAs and AGEs include amino acids, glucose, creatine, and creatinine. By establishing a Pearson’s linear relationship model, the correlation between precursors, protein, fat oxidation and HAAs, AGEs were analyzed. Table 5 shows that the HAA and AGE production is closely related to the TBARS, carbonyl, and precursors. A correlation analysis showed that the formation of HAAs and AGEs was involved in the sharing and competition of some precursors. Norharman was significantly negatively correlated with glucose (*P* < 0.05) and positively correlated to TBARS (*P* < 0.05); Harman was significantly positively correlated to carbonyl (*P* < 0.01) and TBARS (*P* < 0.05) and was negatively correlated with glucose (*P* < 0.05). CML was significantly positively correlated with TBARS (*P* < 0.01) and carbonyl (*P* < 0.05).

In our research, we found that lipid oxidation is closely related to the formation of HAAs and AGEs. For HAAs, many studies have shown that lipid oxidation generates large amounts of free radicals and intermediates, which is conducive to the formation of HAAs [44]. Free radicals can attack amino acids produce active carbonyl products (acetaldehyde and α-ketonic acids) which are key products of Maillard reactions [45]. For AGEs, CML and CEL can be generated in meat products through Maillard reactions and the lipid oxidation pathway [46]. Yu et al. [47] have observed that during the storage of Chinese-style sausages, lipid oxidation leads to an increase in free radicals, which contributes to CML and CEL generation, and lipid peroxidation (the acetol pathway) is also closely related to AGE generation. Yim et al. [48] pointed out that amino acids and methylglyoxal can generate free radicals, thereby promoting the generation of AGEs. In addition to this, lipid oxidation also produces a large number of highly active glycosyl compounds, which react with amino acids or amino residues on proteins to generate CEL and CML [46].

Raw meat undergoes a certain degree of protein denaturation during frozen storage, including protein hydrolysis and oxidation, which can provide precursors (partially increased free amino acid content) and intermediates (oxidation to produce aldehydes) for HAA and AGE production. Protein oxidation will change the structure of raw meat protein, which may expose some specific binding sites of HAAs and AGEs and promote the production of related HAAs and AGEs. At the same time, the formation of ice crystals during freezing causes cells to rupture and release related enzymes. According to the formation mechanism of protein-bound HAAs, the reason for the accumulation of protein-bound HAAs during freezing may be related to the protein structure unfolding during freezing. The exposed amino groups of the protein may react with related precursors, and protein aggregation enhances Norharman and protein adsorption.

The free amino acids and glucose in meat provide superior conditions for the generation of HAAs and AGEs. They can promote the production of HAAs and AGEs by participating in the Maillard reaction. In studying the effects of sugars on HAAs, Tai et al. [36] found that while the formation of HAAs is promoted, the consumption of amino acids also increases significantly in the system. Fekkes et al. [49] have shown that the production of Norharman and Harman in animals is proportional to the intake of tryptophan, which is an important precursor. In addition, creatine and creatinine are the precursors of a variety of HAAs, studies have shown that adding creatine and creatinine to the system will significantly promote the production of heterocyclic amines [38].

## 3. Materials and Methods

### 3.1. Chemicals and Materials

All of the reagents were of analytical grade, unless otherwise stated. The CML standard (purity level: >98%), CEL standard (purity level: >98%), d4-CML isotope internal standard (purity level: >98%), and d4-CEL isotope (purity level: >98%) were purchased from Santa Cruz Biotechnology (Dallas, TX, USA). Harman and Norharman standards (purity level: >98%) were purchased from Santa Cruz Biotechnology (Shanghai, China) Co., Ltd.

### 3.2. Preparing Meat

The whole longissimus lumborum (LL, h postmortem; the pig was approximately 6 months old, and the average carcass weight was approximately 79.6 kg) were obtained from four pig carcasses, purchased from a local market (Wuxi, China) and shipped to the laboratory in crushed ice. Visible fat and connective tissue were removed. Each sample was sliced 2 cm thick and sealed in moisture-impermeable polyethylene bags. After freezing at −20 °C for 24 h, it was thawed at 4 °C for 6 h, which was a freeze–thaw cycle. The number of freeze–thaw cycles varied by group, from 0 to 7 times, divided into five groups: freeze and thaw 0, 1, 3, 5, and 7 times. At least 10 samples were used for each treatment.

### 3.3. Determination of Lipid Oxidation

#### 3.3.1. Determination of TBARS

The TBARS content was determined by the methods used in a previous study [50], with small modifications. First, 2.0 g of the freeze-dried sample were added to a 3 mL thiobarbituric acid solution (1 g thiobarbituric acid dissolved in 75 mL of a 0.1 mol/L NaOH solution and diluted to 100 mL with water) and 17 mL of a TCA–HCl (50 mL 25% trichloroacetic acid solution and 30 mL of a 0.6 mol/L HCl solution mixed with water to 500 mL), which was homogenized and transferred to a 45 mL pressure bottle, and then filled with nitrogen and boiled in water for one hour. Next, 5 mL of the supernatant were mixed with 5 mL chloroform and centrifuged at 1800× *g* for 15 min. The supernatant was measured at an absorbance of 532 nm.

TBARS (mg/kg) = A_532_/W_S_ × 9.48, where A_532_ and W_S_ represent the absorbance at 532 nm and the weight (g) of the sample, respectively, with “9.48” used as a constant.

#### 3.3.2. Determination of Free Radicals

To determine the free radicals, 80.0 mg of the lyophilized sample were put into a nuclear magnetic tube, and its free radical content was measured using an EMXplus-10/12 electron spin resonance spectrometer. The instrument parameters were set as follows: center magnetic field strength, 3350.00 G; scan width, 200 G; scan time, 60.00 s; microwave energy, 20 mW; and adjustment amplitude, 3.000 G. The free radical content was expressed as the difference between the highest peak and the lowest peak.

#### 3.3.3. Determination of Fatty Acids

To determine the fatty acids, 0.5 g of the sample were weighed into a flask, and 1 mL undecanoic acid internal standard and 10 mL chloroform/methanol solution (*v*:*v* 2:1)were added. After soaking for 24 h, the sample was filtered from the solution in a 25 mL graduated tube, and this step was repeated twice. After evaporation in a water bath at 70 °C, 2 mL of 0.5 mol/L NaOH methanol solutions were added and heated in a water bath at 60 °C, until the oil beads completely dissolved. After cooling, 2 mL of a 25% boron trifluoride diethyl ether solution were added and esterified at 60 °C for 30 min. After cooling, 2 mL of n-hexane were added, and the solution was mixed again. Then, 2 mL of the saturated sodium chloride solution were added. After centrifugation, the supernatant was used for gas chromatography in a SP-2560 capillary column (100 m × 0.25 mm, 0.2 mm). The carrier gas was N_2_; the split ratio was 100:1; the injection volume was 1 microliter; the inlet temperature was 270 °C; and the detector temperature was 280 °C. The program temperature cycled as follows: maintained at 100 °C for 13 min, increased at a rate of 10 °C/min to 180 °C, maintained for 6 min; increased at a rate of 1 °C/min to 190 °C, maintained for 12 min; increased at a rate of 4 °C/min to 230 °C, and maintained for 18 min.

### 3.4. Determination of Protein Oxidation

#### 3.4.1. Determination of Carbonyl

The carbonyl content was detected by the 2,4-dinitrophenyl-hydrazine (DNPH) colorimetric method, as reported by Levine et al. [51]. Muscle samples were homogenized in 10 volumes (*w*/*v*) of ultrapure water. The homogenate was mixed with 5 volumes (*v*/*v*) DNPH in 2 M HCl, and the same volume of 2 M HCl was added to the blank, incubated at room temperature for 60 min. Two microliters of 20% trichloroacetic acid (TCA) was added and centrifuged at 10,000× *g* for 10 min. The precipitate was washed with 4 mL of the ethanol:ethyl acetate (*v*/*v*: 1:1) mixture and repeated three times. Then, the precipitate was blown dry with N_2_, and 1.5 mL 6 M guanidine hydrochloride (dissolved in 20 mmol/L KH_2_PO_4_, pH 6.5) was added, incubated at 37 °C for 40 min. After that, the absorbances were measured at 370 and 280 nm. The carbonyl content was calculated as nanomoles per milligram of protein [52]:

(1) $$\mathrm{Carbonyl}\;\mathrm{content}\;(\mathrm{nmol}/\mathrm{mg}\;\mathrm{protein})\;=\;\frac{{\mathrm{Abs}}_{370}{\;-\;\mathrm{Abs}}_{370}\;(\mathrm{blank})}{{22,000\;\times \;[\mathrm{Abs}}_{280}{\;-\;(\mathrm{Abs}}_{370}{\;-\;\mathrm{Abs}}_{370}\left(\mathrm{blank}\right)\;\times \;0.43]}{\times 10}^{6}$$

#### 3.4.2. Determination of Free Amino Acids

To determine the free amino acids, 1.0 g of the muscle sample was packed in a 50 mL centrifuge tube for freeze-drying to prevent juice from flowing out, and 25 mL of 5% trichloroacetic acid were added. After standing for 2 h, the supernatant was filtered to obtain an amino acid solution, which was then tested after precolumn derivatization. The derivatization step was as follows. First, 200 μL of the amino acid sample solution were obtained, which 20 μL leucine internal standard solution, 100 μL triethylamine acetonitrile solution, and 100 μL of the phenyl isothiocyanate acetonitrile solution was added to. After standing for 1 h, 400 μL of n-hexane were added, and the lower solution was filtered by a 0.22 μm membrane. The test was carried out using a Waters 2695 tandem Waters UV 2487 detector, Venusil-AA column for amino acid analysis (100A, 4.6 mm × 250 mm, 5 μm), a detection wavelength of 254 nm, a column temperature of 40 °C, mobile phase A (15.2 g anhydrous sodium acetate, 1850 mL water, acetonitrile 140 mL, pH 6.5) and mobile phase B (80% acetonitrile solution), an injection volume of 2 μL, a flow rate of 1 mL/min, and gradient elution (0–14 min, 20% B; 14–33 min, 34% B; 33.02–41 min, 100% B; 41.02–49 min, 0% B).

### 3.5. Determination of the Glucose, Creatine, and Creatinine

The glucose, creatine, and creatinine contents were determined by the Ultra-Performance Liquid Chromatography (UPLC^®^) Technology system equipped with a triple quadrupole mass spectrometer (Waters, Milford, MA). The specific instrument parameters are described in Nan et al. [53]. Then, 5 g of the sample were added to 40 mL ultrapure water for the ultrasonic extraction of the supernatant for 30 min and then centrifuged. Next, 0.6 mL of the supernatant were mixed with 1.4 mL absolute ethanol and centrifuged. The supernatant was placed in a liquid vial for testing. The parent ion, daughter ion, cone voltage, collision voltage, and residence time of creatine, creatinine and glucose in multireaction monitoring (MRM) mode are shown in Table S1, Supplementary Materials.

### 3.6. Determination of HAAs and AGEs

#### 3.6.1. Determination of HAAs

To determine the HAAs, we first extracted the total HAAs using an acid hydrolysis method based on a previous study [54]. The sample was hydrolyzed with 6 mol/L hydrochloric acid at 110 °C for 24 h. The HHA extract was diluted to an appropriate multiple, of which 10 mL were loaded onto a Waters Oasis MCX solid-phase extraction cartridge. The solid-phase extraction was carried out as follows: activation (6 mL each of chromatographic methanol, ultrapure water, and 0.1 mol/L hydrochloric acid), loading (all sample solutions), rinsing (6 mL each of 0.1 mol/L HCL and chromatographic methanol), and eluting to collect the HAAs (the volume ratio (*v*/*v*) of methanol:ammonia = 19:1, 6 mL). After drying the eluent with nitrogen, the HAAs were reconstituted with 500 μL chromatographic methanol, filtered with a 0.22 μm filter membrane and then determined.

We then identified and quantified the HAAs using a UPLC system equipped with a triple quadrupole mass spectrometer (Waters, Milford, MA) following the methods of a previous study [55]. The chromatographic column was a CORTECS C18 UPLC column (2.1 mm × 150 mm, 1.6 μm), and the column temperature was 45 °C. Mobile phase: phase A was 100% acetonitrile; phase B was 3 mmol/L ammonium acetate solution (pH: 4.76); and the flow rate was 0.3 mL/min. The gradient elution was performed as follows: 0–0.1 min, 40% A; 0.1–18 min, 40% A; 18–20 min, 70% A; 20–22 min, 100% A; 22–22.1 min, 4% A. The injection volume of free HAs was 5 μL, and the injection volume of bound HAs was 3 μL.

Mass spectrometry conditions: MRM; ionization method: electrospray (ESI) positive ion mode; ion source temperature: 120 °C; desolation temperature: 360 °C; flow rate: 680 L/h; capillary voltage: 3.5 kV; cone gas: nitrogen gas with a flow rate of 55 L/h; collision gas: argon gas with a flow rate of 0.13 mL/min; scanning range: 2–2000 Da.

#### 3.6.2. Determination of AGEs

To determine the AGEs, we first prepared the samples intended for CML and CEL analysis according to the method described by He et al. [56] and Yu et al. [57]. The samples were accurately weighed and then freeze-dried and crushed with a 5 mg protein equivalent and defatted with n-hexane (5 mL for 3 times). The solvent was evaporated and transferred to a 15 mL thick-walled pressure bottle, and 1 mL of the NaBH_4_ solution and 1.5 mL of 0.2 M borate buffer (pH: 9.2) were added. 1-Octanol was added to defoam and reduced overnight at 4 °C. Then, 2.5 mL concentrated hydrochloric acid was added and digested at 110 °C for 24 h under nitrogen protection. After digestion, the digestion solution was diluted to 10 mL. After filtering, 300 μL of the digestion solution was separated and blown dry with nitrogen. Then, 150 μL of an internal standard (1.0 μg/mL d_4_-CML and d_4_-CEL mixed standard) were added, with 2 mL ultrapure water. After the water was reconstituted, it was passed through a solid-phase extraction column.

A Waters Oasis MCX solid-phase extraction cartridge was used for solid-phase extraction. The solid-phase extraction method was used as follows. In the activation stage, 3 mL chromatographic methanol and 3 mL 0.1 mol/L HCl solution were used for activation. The reconstituted sample solution was loaded. During the elution phase, 3 mL of a 0.1 mol/L HCl solution and 3 mL of a methanol ammonia solution (the volume ratio (*v*/*v*) of methanol:ammonia = 19:1) were used for elution. Finally, the eluate was blown dry with nitrogen, reconstituted with 300 μL water and filtered with a 0.22 μm water filter to obtain the AGE test solution.

The contents of AGEs (CML and CEL) were determined using an ACQUITY UPLC TQD ultrahigh-performance liquid chromatography tandem triple quadrupole mass spectrometer. The specific detection conditions for each test were as follows. Chromatography: the Waters chromatographic column was T3 (2.1 mm × 150 mm, 1.7 μm); the column temperature was 45 °C; the sample chamber was 4 °C; mobile phase A: methanol, mobile phase B: 0.1% formic acid in water; the flow rate was 0.200 mL/min; the gradient elution was 0.10 min, 1% A; 1.00 min, 3% A; 5.00 min, 50% A; 7.00 min to 9.00 min, 100% A; 10.00 min, 1.0% A; the injection volume was 2 μL. The conditions for mass spectrometry were ESI as an ion source using the positive ion acquisition mode and technology. The temperature parameters were 110 °C and 400 °C for the ion source and dissolvent gas, respectively; nitrogen was used for cone gas; the flow rate was 50 L/h; argon gas was used for collision gas; the flow rate was 0.13 mL/min; the capillary voltage was 3.5 kV, and the scanning range was 2–2000 Da.

### 3.7. Statistical Analysis

Harman, Norharman, CML, CEL contents of meat (freeze-thawed 0, 1, 3, 5, and 7 times) were related to TBARS, carbonyl, glucose, creatine, and creatinine contents of meat, respectively, using the Pearson correlation coefficient model. The results of the normal distribution test were significant at a 0.05 level, and the data followed a normal distribution. Three independent repeated experiments were prepared for all analyses, and the results were expressed as mean ± standard deviation (SPSS Statistics 22). The liquid and mass spectral data were processed using a Mass Lynx V4.1 workstation. Significant differences were detected using Statistix9 analysis (following the general linear model and using the least-significant difference method); and the charts were drawn using Origin 8.0.

## 4. Conclusions

In conclusion, during freeze–thaw cycles, HAAs and AGEs increased significantly. Freeze–thaw treatment accelerated the oxidation of lipids and proteins and promoted the movement of precursors. The contents of TBARS, protein carbonyl, and free radicals increased, and the precursors content decreased with increasing freeze–thaw cycles. Oxidation can generate large amounts of free radicals, and the movement of precursors can promote the Maillard reaction, which all promoted the formation of HAAs and AGEs. The correlation of oxidation (proteins and lipids), precursors (glucose, creatine, and creatinine), and end products (HAAs and AGEs) was analyzed, and end products had a significant relationship with oxidation indicators and glucose. Because of the harmful effects of HAAs and AGEs on human health, such as the carcinogenicity of HAAs, AGEs are associated with many chronic diseases. We need to avoid repeated freezing and thawing and reduce the occurrence of oxidation in actual production, transportation, and retail.

## Figures and Tables

**Table 1:** The content of fatty acid (%) in raw meat during frozen storage.

Freeze–Thaw Cycles	Control	FT-1	FT-3	FT-5	FT-7
C14:0	1.26 ± 0.02 ^a^	1.26 ± 0.09 ^a^	1.26 ± 0.04 ^a^	1.26 ± 0.07 ^a^	1.17 ± 0.06 ^b^
C16:0	22.14 ± 0.82 ^a^	21.96 ± 0.64 ^a^	20.07 ± 0.80 ^a,b^	20.25 ± 0.80 ^a,b^	19.89 ± 0.83 ^b^
C18:0	12.87 ± 1.28 ^a^	11.78 ± 0.25 ^a,b^	10.26 ± 0.51 ^b^	10.80 ± 0.07 ^b^	10.62 ± 0.15 ^b^
C18:1	40.68 ± 3.48 ^a^	37.98 ± 2.14 ^a^	38.61 ± 3.05 ^a^	38.61 ± 1.91 ^a^	38.16 ± 2.47 ^a^
C16:1	3.80 ± 0.12 ^a^	3.98 ± 0.13 ^a^	3.06 ± 0.19 ^b^	2.79 ± 0.20 ^c^	2.79 ± 0.20 ^c^
C20:1	1.26 ± 0.02 ^a^	0.99 ± 0.07 ^b^	0.99 ± 0.06 ^b^	0.99 ± 0.04 ^b^	1.08 ± 0.14 ^b^
C18:2	15.40 ± 0.66 ^a^	14.03 ± 0.69 ^a^	8.46 ± 0.50 ^b^	8.46 ± 0.12 ^b^	8.46 ± 0.20 ^b^
C18:3	0.45 ± 0.07 ^a^	0.45 ± 0.03 ^a^	0.45 ± 0.07 ^a^	0.45 ± 0.06 ^a^	0.45 ± 0.07 ^a^
Saturated fatty acid	36.27 ± 0.96 ^a^	35.91 ± 1.28 ^a,b^	31.59 ± 2.14 ^b^	32.31 ± 1.18 ^b^	31.77 ± 0.68 ^b^
Monounsaturated fatty acid	43.74 ± 1.17 ^a^	40.95 ± 1.01 ^b^	42.66 ± 0.82 ^a,b^	42.39 ± 0.89 ^a,b^	42.03 ± 1.26 ^a,b^
Polyunsaturated fatty acids	15.85 ± 0.90 ^a^	15.48 ± 0.71 ^a^	8.91 ± 1.09 ^b^	8.91 ± 1.17 ^b^	8.91 ± 1.04 ^b^

Comparisons were made within the same line. Data were presented as means ± standard deviations (*n* = 3). Different letters (a–d) in the same group represent significant difference (*P* < 0.05).

**Table 2:** The content of free amino acid (mg/g) in pork during frozen storage.

Freeze–Thaw Cycles	0	1	3	5	7
Asp	0.01 ± 0.00 ^a^	0.01 ± 0.00 ^a^	0.01 ± 0.00 ^a^	0.01 ± 0.00 ^a^	0.01 ± 0.00 ^a^
Ser	0.09 ± 0.01 ^a^	0.11 ± 0.01 ^a^	0.12 ± 0.02 ^a^	0.12 ± 0.01 ^a^	0.11 ± 0.01 ^a^
Gly	0.18 ± 0.02 ^b^	0.22 ± 0.01 ^a,b^	0.23 ± 0.02 ^a,b^	0.26 ± 0.02 ^a^	0.27 ± 0.03 ^a^
His	1.21 ± 0.10 ^b^	1.33 ± 0.09 ^a,b^	1.34 ± 0.10 ^a,b^	1.50 ± 0.07 ^a^	1.51 ± 0.09 ^a^
Arg	3.81 ± 0.10 ^b^	4.19 ± 0.08 ^a,b^	4.21 ± 0.15 ^a,b^	4.24 ± 0.20 ^a,b^	4.41 ± 0.10 ^a^
Thr	1.88 ± 0.10 ^b^	2.10 ± 0.20 ^a,b^	1.98 ± 0.13 ^a,b^	2.28 ± 0.19 ^a^	2.37 ± 0.10 ^a^
Pro	0.02 ± 0.00 ^a^	0.02 ± 0.00 ^a^	0.02 ± 0.00 ^a^	0.02 ± 0.00 ^a^	0.02 ± 0.00 ^a^
Ala	0.89 ± 0.03 ^b^	0.91 ± 0.01 ^a,b^	0.91 ± 0.02 ^a,b^	0.94 ± 0.01 ^a^	0.95 ± 0.01 ^a^
Tyr	0.04 ± 0.00 ^a^	0.04 ± 0.00 ^a^	0.04 ± 0.00 ^a^	0.04 ± 0.00 ^a^	0.04 ± 0.00 ^a^
Val	0.08 ± 0.00 ^a^	0.08 ± 0.00 ^a^	0.08 ± 0.00 ^a^	0.08 ± 0.00 ^a^	0.08 ± 0.00 ^a^
Met	0.12 ± 0.01 ^b^	0.13 ± 0.00 ^a,b^	0.13 ± 0.01 ^a,b^	0.12 ± 0.00 ^a,b^	0.14 ± 0.00 ^a^
Ile	0.10 ± 0.01 ^b^	0.12 ± 0.02 ^a,b^	0.13 ± 0.01 ^a,b^	0.14 ± 0.02 ^a,b^	0.19 ± 0.02 ^a^
Phe	0.17 ± 0.04 ^b^	0.16 ± 0.02 ^b^	0.21 ± 0.04 ^a,b^	0.24 ± 0.01 ^a^	0.23 ± 0.02 ^a^
Lys	0.23 ± 0.01 ^b^	0.21 ± 0.01 ^b^	0.25 ± 0.02 ^a,b^	0.27 ± 0.02 ^a,b^	0.31 ± 0.01 ^a^
Total free amino acids	9.73 ± 0.02 ^a^	9.81 ± 0.01 ^b^	9.84 ± 0.02 ^c^	10.44 ± 0.01 ^c^	10.82 ± 0.02 ^d^

Comparisons were made within the same line. Data were presented as means ± standard deviations (*n* = 3). Different letters (a–d) in the same group represent significant difference (*P* < 0.05).

**Table 3:** The glucose, creatine, and creatinine (μg/g) values of raw meat during frozen storage.

Freeze–Thaw Cycles	Glucose	Creatine	Creatinine
0	29.19 ± 1.15 ^a^	5122.05 ± 159.07 ^a^	89.13 ± 4.18 ^a^
1	26.82 ± 2.97 ^a^	4296.26 ± 306.48 ^c^	85.29 ± 4.21 ^b^
3	18.35 ± 3.21 ^b^	4407.15 ± 571.86 ^c^	82.75 ± 3.61 ^b^
5	20.60 ± 3.72 ^b^	4605.81 ± 331.16 ^b,c^	90.43 ± 4.59 ^a^
7	19.29 ± 1.41 ^b^	4818.18 ± 66.08 ^a,b^	88.43 ± 1.33 ^a,b^

Comparisons were made within the same column. Data were presented as means ± standard deviations (*n* = 3). Different letters (a–c) in the same group represent significant difference (*P* < 0.05).

**Table 4:** The heterocyclic aromatic amines (HAAs; ng/g dry basis) and advanced glycation end products (AGEs; ng/g dry basis) values of raw meat during freeze–thaw cycles.

Cycles	Norharman	Harman	CEL	CML
0	116.03 ± 8.24 ^b^	88.12 ± 8.90 ^a^	34.56 ± 2.23 ^b^	36.23 ± 4.32 ^b^
1	125.43 ± 9.12 ^a,b^	90.06 ± 8.40 ^a^	35.25 ± 1.32 ^b^	28.51 ± 5.49 ^b^
3	137.18 ± 9.00 ^a^	92.67 ± 9.13 ^a^	35.98 ± 2.56 ^b^	42.09 ± 6.42 ^a,b^
5	138.53 ± 5.96 ^a^	95.03 ± 9.38 ^a^	37.87 ± 3.09 ^a,b^	53.58 ± 9.62 ^a^
7	139.62 ± 6.61 ^a^	102.81 ± 14.59 ^a^	38.64 ± 3.24 ^a^	41.31 ± 3.05 ^a,b^

Comparisons were made within the same column. Data were presented as means ± standard deviations (*n* = 3). Different letters (a–b) in the same group represent significant difference (*P* < 0.05).

**Table 5:** Correlation coefficient between hazards and TBARS, carbonyl, precursors in raw meat.

	TBARS	Carbonyl	Glucose	Creatine	Creatinine
**Norharman**	0.910 *	0.849	−0.914 *	−0.403	−0.083
**Harman**	0.951 *	0.990 **	−0.920*	0.050	0.229
**CML**	0.629	0.417	−0.509	0.115	0.444
**CEL**	0.992 **	0.933*	−0.727	0.285	0.219

** *P* < 0.01; * *P* < 0.05.

## Data Availability

Data are contained within the article and the Supplementary Materials.

## Supplementary Materials

The following are available online. Table S1: MS/MS parameters and characteristic ions of creatine, creatinine, and glucose, Table S2: MS/MS parameters and characteristic ions of Harman and Norharman, Table S3: MS/MS parameters and characteristic ions of AGEs and its internal standard.

## References

[1] Pietrasik Z., Janz J.A.M. (2009). Influence of freezing and thawing on the hydration characteristics, quality, and consumer acceptance of whole muscle beef injected with solutions of salt and phosphate. Meat Sci..

[2] Ali S., Zhang W., Rajput N., Khan M.A., Li C.-B., Zhou G.-H. (2015). Effect of multiple freeze–thaw cycles on the quality of chicken breast meat. Food Chem..

[3] Kim G.-D., Jung E.-Y., Lim H.-J., Yang H.-S., Joo S.-T., Jeong J.-Y. (2013). Influence of meat exudates on the quality characteristics of fresh and freeze-thawed pork. Meat Sci..

[4] Coombs C.E.O., Holman B.W.B., Friend M.A., Hopkins D.L. (2017). Long-term red meat preservation using chilled and frozen storage combinations: A review. Meat Sci..

[5] Jeong J.Y., Kim G.D., Yang H.S., Joo S.T. (2011). Effect of freeze-thaw cycles on physicochemical properties and color stability of beef semimembranosus muscle. Food Res. Int..

[6] Srinivasan S., Xiong Y.L., Blanchard S.P., Tidwell J.H. (1997). Physicochemical changes in prawns (Machrobrachium rosenbergii) subjected to multiple freeze-thaw cycles. J. Food Sci..

[7] Xia X.F., Kong B.H., Liu Q., Liu J. (2009). Physicochemical change and protein oxidation in porcine longissimus dorsi as influenced by different freeze-thaw cycles. Meat Sci..

[8] Benjakul S., Visessanguan W., Thongkaew C., Tanaka M. (2003). Comparative study on physicochemical changes of muscle proteins from some tropical fish during frozen storage. Food Res. Int..

[9] Jaegerstad M., Laser Reuterswaerd A., Oeste R., Dahlqvist A., Grivas S., Olsson K., Nyhammar T. (1983). Creatinine and Maillard reaction products as precursors of mutagenic compounds formed in fried beef. Mail. React. Foods Nutr..

[10] Agim Z.S., Cannon J.R. (2018). Alterations in the nigrostriatal dopamine system after acute systemic PhIP exposure. Toxicol. Lett..

[11] Cruz-Hernandez A., Agim Z.S., Montenegro P.C., McCabe G.P., Rochet J.C., Cannon J.R. (2018). Selective dopaminergic neurotoxicity of three heterocyclic amine subclasses in primary rat midbrain neurons. Neurotoxicology.

[12] Kosmopoulos M., Drekolias D., Zavras P.D., Piperi C., Papavassiliou A.G. (2019). Impact of advanced glycation end products (AGEs) signaling in coronary artery disease. Biochim. Biophys. Acta-Mol. Basis Dis..

[13] Nowotny K., Schroter D., Schreiner M., Grune T. (2018). Dietary advanced glycation end products and their relevance for human health. Ageing Res. Rev..

[14] Lipeng H., Lin L., Bing L., Di Z., Yuting L., Zhenbo X., Guoqin L. (2013). Hydroxyl radical induced by lipid in Maillard reaction model system promotes diet-derived Nepsilon-carboxymethyllysine formation. Food Chem. Toxicol..

[15] Zamora R., Hidalgo F.J. (2020). Formation of heterocyclic aromatic amines with the structure of aminoimidazoazarenes in food products. Food Chem..

[16] Park S.Y., Yoo S.S., Uh J.H., Eun J.B., Lee H.C., Kim Y.J., Chin K.B. (2007). Evaluation of lipid oxidation and oxidative products as affected by pork meat cut, packaging method, and storage crime during frozen storage (-10 degrees C). J. Food Sci..

[17] Pan N., Dong C., Du X., Kong B., Sun J., Xia X. (2021). Effect of freeze-thaw cycles on the quality of quick-frozen pork patty with different fat content by consumer assessment and instrument-based detection. Meat Sci..

[18] Leygonie C., Britz T.J., Hoffman L.C. (2012). Impact of freezing and thawing on the quality of meat: Review. Meat Sci..

[19] Sun Q.X., Chen Q., Xia X.F., Kong B.H., Diao X.P. (2019). Effects of ultrasound-assisted freezing at different power levels on the structure and thermal stability of common carp (*Cyprinus carpio*) proteins. Ultrason. Sonochem..

[20] Fu M.X., Requena J.R., Jenkins A.J., Lyons T.J., Baynes J.W., Thorpe S.R.J.J.O.B.C. (1996). The Advanced Glycation End Product,N-(Carboxymethyl)lysine, Is a Product of both Lipid Peroxidation and Glycoxidation Reactions. J. Biol. Chem..

[21] Yu L., Gao C., Zeng M., He Z., Wang L., Zhang S., Chen J. (2016). Effects of raw meat and process procedure on Nε-carboxymethyllysine and Nε-carboxyethyl-lysine formation in meat products. Biotechnology.

[22] Jensen P.N., Danielsen B., Bertelsen G., Skibsted L.H., Andersen M.L. (2005). Storage stabilities of pork scratchings, peanuts, oatmeal and muesli: Comparison of ESR spectroscopy, headspace-GC and sensory evaluation for detection of oxidation in dry foods. Food Chem..

[23] Hernández P., Navarro J.L., Toldrá F. (1999). Effect of frozen storage on lipids and lipolytic activities in the longissimus dorsi muscle of the pig. Z Lebensm. Unters..

[24] Igene J.O., Pearson A.M., Dugan L.R., Price J.F. (1980). Role of triglycerides and phospholipids on development of rancidity in model meat systems during frozen storage. Food Chem..

[25] Estevez M. (2011). Protein carbonyls in meat systems: A review. Meat Sci..

[26] Fábio A.P.S., Estevez M., Valquíria C.S.F., Samara A.S., Leanderson T.M.L., Elza I.I., Massami S., Marta S.M. (2018). Protein and lipid oxidations in jerky chicken and consequences on sensory quality. LWT.

[27] Abbasi E., Sarteshnizi R.A., Gavlighi H.A., Nikoo M., Azizi M.H., Sadeghinejad N. (2019). Effect of partial replacement of fat with added water and tragacanth gum (*Astragalus gossypinus* and *Astragalus compactus*) on the physicochemical, texture, oxidative stability, and sensory property of reduced fat emulsion type sausage. Meat Sci..

[28] Sher A., Wangang Z., Nasir R., Ammar Khan M., Chun-Bao L., Guang-Hong Z. (2015). Effect of multiple freeze-thaw cycles on the quality of chicken breast meat. Food Chem..

[29] Monti S.M., Ritieni A., Sacchi R., Skog K., Borgen E., Fogliano V. (2001). Characterization of phenolic compounds in virgin olive oil and their effect on the formation of carcinogenic/mutagenic heterocyclic amines in a model system. J. Agric. Food Chem..

[30] Morita H., Noda K., Umeda M. (1985). Mutagenicities of nickel and cobalt compounds in a mammalian cell line. Mutat. Res. /Environ. Mutagenesis Relat. Subj..

[31] Szterk A., Roszko M., Malek K., Kurek M., Zbiec M., Waszkiewicz-Robak B. (2012). Profiles and concentrations of heterocyclic aromatic amines formed in beef during various heat treatments depend on the time of ripening and muscle type. Meat Sci..

[32] Kanzler C., Schestkowa H., Haase P.T., Kroh L.W. (2017). Formation of Reactive Intermediates, Color, and Antioxidant Activity in the Maillard Reaction of Maltose in Comparison to D-Glucose. J. Agric. Food Chem..

[33] Solyakov A., Skog K. (2002). Screening for heterocyclic amines in chicken cooked in various ways. Food Chem. Toxicol..

[34] Utrera M., Parra V., Estevez M. (2014). Protein oxidation during frozen storage and subsequent processing of different beef muscles. Meat Sci..

[35] Villaverde A., Estevez M. (2013). Carbonylation of Myofibrillar Proteins through the Maillard Pathway: Effect of Reducing Sugars and Reaction Temperature. J. Agric. Food Chem..

[36] Tai C.Y., Lee K.H., Chen B.H. (2001). Effects of various additives on the formation of heterocyclic amines in fried fish fibre. Food Chem..

[37] Zeng M.M., Li Y., He Z.Y., Qin F., Chen J. (2014). Effects of Different Precursors on Formation of Heterocyclic Amines of Processed Food by Ultra-high Performance Liquid Chromatography Tandem Mass Spectrometry combined with Principal Component Analysis. Chin. J. Anal. Chem..

[38] Lee H.I., Lin M.Y., Chan S.C. (1994). Formation And Identification Of Carcinogenic Heterocyclic Aromatic-Amines In Boiled Pork Juice. Mutat. Res..

[39] Gibis M., Weiss J. (2017). Inhibitory effect of cellulose fibers on the formation of heterocyclic aromatic amines in grilled beef patties. Food Chem..

[40] Persson E., Sjöholm I., Nyman M., Skog K. (2004). Addition of various carbohydrates to beef burgers affects the formation of heterocyclic amines during frying. J. Agric. Food Chem..

[41] Ka-Wing C., Feng C., Mingfu W. (2006). Heterocyclic amines: Chemistry and health. Mol. Nutr. Food Res..

[42] Zhu Z., Huang S., Khan I.A., Cheng Y., Yu Y., Zhang C., Huang J., Huang M., Zhou X. (2019). The effect of oxidation and Maillard reaction on formation of Nε -carboxymethyllysine and Nε-carboxyethyllysine in prepared chicken breast. Cytajournal Food.

[43] Yang G., Huang Y., Wu X., Lin X., Xu J., Chen X., Bai X., Li Q. (2018). Endogenous Secretory Receptor for Advanced Glycation End Products Protects Endothelial Cells from AGEs Induced Apoptosis. Biomed Res. Int..

[44] Sanz-Alaejos M., Afonso A.M. (2011). Factors that affect the content of heterocyclic aromatic amines in foods. Compr. Rev. Food Sci. Food Saf..

[45] Zheng J., Bizzozero O.A. (2010). Traditional reactive carbonyl scavengers do not prevent the carbonylation of brain proteins induced by acute glutathione depletion. Free Radic. Res..

[46] Miyata T., Kurokawa K., De Strihou C.V. (2000). Advanced glycation and lipoxidation end products: Role of reactive carbonyl compounds generated during carbohydrate and lipid metabolism. J. Am. Soc. Nephrol..

[47] Yu L.G., Chai M., Zeng M.M., He Z.Y., Chen J. (2018). Effect of lipid oxidation on the formation of N-epsilon-carboxymethyl-lysine and N-epsilon-carboxyethyl-lysine in chinese-style sausage during storage. Food Chem..

[48] Yim H.S., Kang S.O., Hah Y.C., Chock P.B., Yim M.B. (1995). Free-Radicals Generated During the Glycation Reaction of Amino-Acids By Methylglyoxal - A Model Study Of Protein-Cross-Linked Free-Radicals. J. Biol. Chem..

[49] Fekkes D., Tuiten A., Bom I., Pepplinkhuizen L. (2001). Tryptophan: A precursor for the endogenous synthesis of norharman in man. Neurosci. Lett..

[50] Sinnhuber R.O., Yu T.C. (1977). The 2-thiobarbituric acid reaction, an objective measure of the oxidative deterioration occurring in fats and oils. J. Jpn. Oil Chem. Soc..

[51] Levine R.L., Williams J.A., Stadtman E.R., Shacter E. (1994). Carbonyl assays for determination of oxidatively modified proteins. Oxyg. Radic. Biol. Syst. Pt. C.

[52] Soglia F., Petracci M., Ertbjerg P. (2016). Novel DNPH-based method for determination of protein carbonylation in muscle and meat. Food Chem..

[53] Nan Z., Liang Z., Chao M., Jian-rong Y., Jian-mei Z. (2016). Changes in free fatty acid, free amino acid, and nucleotide content during preparation of stewed pork. Mod. Food Sci. Technol..

[54] Chen J., He Z.Y., Qin F., Chen J., Zeng M.M. (2017). Formation of Free and Protein-Bound Heterocyclic Amines in Roast Beef Patties Assessed by UPLC-MS/MS. J. Agric. Food Chem..

[55] Diaodiao Y., Zhiyong H., Daming G., Fang Q., Shaolin D., Peng W., Xinglian X., Jie C., Maomao Z. (2019). Effects of smoking or baking procedures during sausage processing on the formation of heterocyclic amines measured using UPLC-MS/MS. Food Chem..

[56] He J.L., Zeng M.M., Zheng Z.P., He Z.Y., Chen J. (2014). Simultaneous determination of N (epsilon)-(carboxymethyl) lysine and N (epsilon)-(carboxyethyl) lysine in cereal foods by LC-MS/MS. Eur. Food Res. Technol..

[57] Yu L.G., He Z.Y., Zeng M.M., Zheng Z.P., Chen J. (2016). Effect of irradiation on N-epsilon-carboxymethyl-lysine and N-epsilon-carboxyethyl-lysine formation in cooked meat products during storage. Radiat. Phys. Chem..

