# Supplementary material for: Effect of Freeze-Thaw Cycles on the Oxidation of Protein and Fat and Its Relationship with the Formation of Heterocyclic Aromatic Amines and Advanced Glycation End Products in Raw Meat
Source: Molecules. 2021 Feb 26;26(5):1264. doi: 10.3390/molecules26051264 (PMC7956273; doi:10.3390/molecules26051264)
Supplement: Supplementary file 1 [file molecules-26-01264-s001.zip › Supplementary Materials proof.pdf]

Table S1. MS/MS parameters and characteristic ions of creatine, creatinine and glucose.

Name	Parent ion (m/z)	Daughter ion (m/z)	Cone voltage (V)	Collision energy (eV)	Dwell time (s)
Creatine	132	90	28	16	0.10
Creatinine	114	44	34	20	0.10
Glucose	383	203	20	10	0.10

Table S2. MS/MS parameters and characteristic ions of Harman and Norharman.

Name	Parent ion (m/z)	Daughter ion (m/z)	Cone voltage (V)	Collision energy (eV)	Dwell time (s)
Harman	183	115	30	40	0.15
Norharman	169	115	30	25	0.15

Table S3. MS/MS parameters and characteristic ions of AGEs and its internal standard.

Name	Parent ion (m/z)	Daughter ion (m/z)	Cone voltage (V)	Collision energy (eV)	Dwell time (s)
CML	205	84	30	15	0.2
d_4-CML	209	88	30	15	0.2
CEL	219	84	30	30	0.2
d_4-CEL	223	88	30	30	0.2
