# Supplementary material for: Effect of Freeze-Thaw Cycles on the Oxidation of Protein and Fat and Its Relationship with the Formation of Heterocyclic Aromatic Amines and Advanced Glycation End Products in Raw Meat
Source: Molecules. 2021 Feb 26;26(5):1264. doi: 10.3390/molecules26051264 (PMC7956273; doi:10.3390/molecules26051264)
Supplement: Supplementary file 1 [file molecules-26-01264-s001.zip › Table 1 Fatty acids.pdf]

Table 1 The content of fatty acid (%) in pork during frozen storage.

Freeze-thaw cycles	Control	FT-1	FT-3	FT-5	FT-7
C14:0	1.26 ± 0.02[a]	1.26 ± 0.09[a]	1.26 ± 0.04[a]	1.26 ± 0.07[a]	1.17 ± 0.06[b]
C16:0	22.14 ± 0.82[a]	21.96 ± 0.64[a]	20.07 ± 0.80[ab]	20.25 ± 0.80[ab]	19.89 ± 0.83[b]
C18:0	12.87 ± 1.28[a]	11.78 ± 0.25[ab]	10.26 ± 0.51[b]	10.80±0.07[b]	10.62 ± 0.15[b]
C18:1	40.68 ± 3.48[a]	37.98 ± 2.14[a]	38.61 ± 3.05[a]	38.61 ± 1.91[a]	38.16 ± 2.47[a]
C16:1	3.80 ± 0.12[a]	3.98 ± 0.13[a]	3.06 ± 0.19[b]	2.79 ± 0.20[c]	2.79 ± 0.20[c]
C20:1	1.26 ± 0.02[a]	0.99 ± 0.07[b]	0.99 ± 0.06[b]	0.99 ± 0.04[b]	1.08 ± 0.14[b]
C18:2	15.40 ± 0.66[a]	14.03 ± 0.69[a]	8.46 ± 0.50[b]	8.46 ± 0.12[b]	8.46 ± 0.20[b]
C18:3	0.45 ± 0.07[a]	0.45 ± 0.03[a]	0.45 ± 0.07[a]	0.45 ± 0.06[a]	0.45 ± 0.07[a]
saturated fatty acid	36.27 ± 0.96[a]	35.91 ± 1.28[ab]	31.59 ± 2.14[b]	32.31 ± 1.18[b]	31.77 ± 0.68[b]
Monounsaturated fatty acid	43.74 ± 1.17[a]	40.95 ± 1.01[b]	42.66 ± 0.82[ab]	42.39 ± 0.89[ab]	42.03 ± 1.26[ab]
Polyunsaturated fatty acids	15.85 ± 0.90[a]	15.48 ± 0.71[a]	8.91 ± 1.09[b]	8.91 ± 1.17[b]	8.91 ± 1.04[b]

[*]Comparisons were made within the same column; Data were presented as means ± standard deviations（n=3）

[a-d]Different letters in the same group represent significant difference (p<0.05)
