# Supplementary material for: Effect of Freeze-Thaw Cycles on the Oxidation of Protein and Fat and Its Relationship with the Formation of Heterocyclic Aromatic Amines and Advanced Glycation End Products in Raw Meat
Source: Molecules. 2021 Feb 26;26(5):1264. doi: 10.3390/molecules26051264 (PMC7956273; doi:10.3390/molecules26051264)
Supplement: Supplementary file 1 [file molecules-26-01264-s001.zip › Table 2 Amino acids.pdf]

Table 2 The content of free amino acid (mg/g) in pork during frozen storage.

Freeze-thaw cycles	0	1	3	5	7
Asp	0.01 ± 0.00[a]	0.01 ± 0.00[a]	0.01 ± 0.00[a]	0.01 ± 0.00[a]	0.01 ± 0.00[a]
Glu	0.00 ± 0.00[a]	0.00 ± 0.00[a]	0.00 ± 0.00[a]	0.00 ± 0.00[a]	0.00 ± 0.00[a]
Ser	0.09 ± 0.01[a]	0.11 ± 0.01[a]	0.12 ± 0.02[a]	0.12 ± 0.01[a]	0.11 ± 0.01[a]
Gly	0.18 ± 0.02[b]	0.22 ± 0.01[ab]	0.23 ± 0.02[ab]	0.26 ± 0.02[a]	0.27 ± 0.03[a]
His	1.21 ± 0.10[b]	1.33 ± 0.09[ab]	1.34 ± 0.10[ab]	1.50 ± 0.07[a]	1.51 ± 0.09[a]
Arg	3.81 ± 0.10[b]	4.19 ± 0.08[ab]	4.21 ± 0.15[ab]	4.24 ± 0.20[ab]	4.41 ± 0.10[a]
Thr	1.88 ± 0.10[b]	2.10 ± 0.20[ab]	1.98 ± 0.13[ab]	2.28 ± 0.19[a]	2.37 ± 0.10[a]
Pro	0.02 ± 0.00[a]	0.02 ± 0.00[a]	0.02 ± 0.00[a]	0.02 ± 0.00[a]	0.02 ± 0.00[a]
Ala	0.89 ± 0.03[b]	0.91 ± 0.01[ab]	0.91 ± 0.02[ab]	0.94 ± 0.01[a]	0.95 ± 0.01[a]
Tyr	0.04 ± 0.00[a]	0.04 ± 0.00[a]	0.04 ± 0.00[a]	0.04 ± 0.00[a]	0.04 ± 0.00[a]
Val	0.08 ± 0.00[a]	0.08 ± 0.00[a]	0.08 ± 0.00[a]	0.08 ± 0.00[a]	0.08 ± 0.00[a]
Met	0.12 ± 0.01[b]	0.13 ± 0.00[ab]	0.13 ± 0.01[ab]	0.12 ± 0.00[ab]	0.14 ± 0.00[a]
Cys	0.00 ± 0.00[a]	0.00 ± 0.00[a]	0.00 ± 0.00[a]	0.00 ± 0.00[a]	0.00 ± 0.00[a]
Leu	0.18 ± 0.00[b]	0.18 ± 0.00[ab]	0.18 ± 0.00[ab]	0.18 ± 0.00[a]	0.18 ± 0.00[a]
Ile	0.10 ± 0.01[b]	0.12 ± 0.02[ab]	0.13 ± 0.01[ab]	0.14 ± 0.02[ab]	0.19 ± 0.02[a]
Phe	0.17 ± 0.04[b]	0.16 ± 0.02[b]	0.21 ± 0.04[ab]	0.24 ± 0.01[a]	0.23 ± 0.02[a]
Lys	0.23 ± 0.01[b]	0.21 ± 0.01[b]	0.25 ± 0.02[ab]	0.27 ± 0.02[ab]	0.31 ± 0.01[a]
Total free amino acids	9.73±0.02[a]	9.81±0.01[b]	9.84±0.02[c]	10.44±0.01[c]	10.82±0.02[d]

[*]Comparisons were made within the same column; Data were presented as means ± standard deviations（n=3）

[a-d]Different letters in the same group represent significant difference (P<0.05)
