# Supplementary material for: Effect of Freeze-Thaw Cycles on the Oxidation of Protein and Fat and Its Relationship with the Formation of Heterocyclic Aromatic Amines and Advanced Glycation End Products in Raw Meat
Source: Molecules. 2021 Feb 26;26(5):1264. doi: 10.3390/molecules26051264 (PMC7956273; doi:10.3390/molecules26051264)
Supplement: Supplementary file 1 [file molecules-26-01264-s001.zip › Table 3 Precursors.pdf]

Table 3 The glucose、creatine and creatinine value of raw meat during frozen storage.

Freeze-thaw cycles	Glucose(ug/g)	Creatine(ug/g)	Creatinine(ug/g)
0	29.19 ± 1.15^{a}	5122.05 ± 159.07^{a}	89.13 ± 4.18^{a}
1	26.82 ± 2.97^{a}	4296.26 ± 306.48^{c}	85.29 ± 4.21^{b}
3	18.35 ± 3.21^{b}	4407.15 ± 571.86^{c}	82.75 ± 3.61^{b}
5	20.60 ± 3.72^{b}	4605.81 ± 331.16^{bc}	90.43 ± 4.59^{a}
7	19.29 ± 1.41^{b}	4818.18 ± 66.08^{ab}	88.43 ± 1.33^{ab}

[*]Comparisons were made within the same column; Data were presented as means ± standard deviations（n=3）

[a-d]Different letters in the same group represent significant difference (*P*<0.05)
