# Supplementary material for: Effect of Freeze-Thaw Cycles on the Oxidation of Protein and Fat and Its Relationship with the Formation of Heterocyclic Aromatic Amines and Advanced Glycation End Products in Raw Meat
Source: Molecules. 2021 Feb 26;26(5):1264. doi: 10.3390/molecules26051264 (PMC7956273; doi:10.3390/molecules26051264)
Supplement: Supplementary file 1 [file molecules-26-01264-s001.zip › Table 4 HAAs and AGEs.pdf]

**Table 4** The HAAs (ng/g Dry basis) and AGEs (ng/g Dry basis) value of raw meat during frozen storage.

| Cycles | Norharman | Harman | Total HAAs | CEL | CML | Total AGEs |
|---|---|---|---|---|---|---|
| 0 | 116.03 ± 8.24$^{b}$ | 88.12 ± 8.90$^{a}$ | 204.15 ± 8.62$^{b}$ | 34.56 ± 2.23$^{b}$ | 36.23 ± 4.32$^{b}$ | 70.79 ± 3.21$^{b}$ |
| 1 | 125.43 ±11.12$^{ab}$ | 90.06 ± 8.40$^{a}$ | 215.49 ± 9.43$^{ab}$ | 35.25 ± 1.32$^{b}$ | 28.51 ± 5.49$^{b}$ | 63.76 ± 4.19$^{b}$ |
| 3 | 137.18 ± 9.00$^{a}$ | 92.67 ± 9.13$^{a}$ | 229.85 ± 9.11$^{a}$ | 35.98 ± 2.56$^{b}$ | 42.09 ± 6.42$^{ab}$ | 78.07 ± 4.96$^{ab}$ |
| 5 | 138.53 ± 5.96$^{a}$ | 95.03 ± 9.38$^{a}$ | 233.56 ± 8.43$^{a}$ | 37.87 ± 3.09$^{ab}$ | 53.58 ± 9.62$^{a}$ | 91.45 ± 6.74$^{a}$ |
| 7 | 139.62 ± 6.61$^{a}$ | 102.81 ± 14.59$^{a}$ | 242.43 ± 11.84$^{a}$ | 38.64 ± 3.24$^{a}$ | 41.31 ± 3.05$^{ab}$ | 79.95 ± 3.19$^{ab}$ |

[*]Comparisons were made within the same column; Data were presented as means ± standard deviations（n=3）

$^{a\text{-}d}$Different letters in the same group represent significant difference (*P*<0.05)
