# Supplementary material for: Effect of Freeze-Thaw Cycles on the Oxidation of Protein and Fat and Its Relationship with the Formation of Heterocyclic Aromatic Amines and Advanced Glycation End Products in Raw Meat
Source: Molecules. 2021 Feb 26;26(5):1264. doi: 10.3390/molecules26051264 (PMC7956273; doi:10.3390/molecules26051264)
Supplement: Supplementary file 1 [file molecules-26-01264-s001.zip › Table 5 Correlation.pdf]

Table 5 Correlation coefficient between hazards and TBARS、carbonyl、precursor in raw meat.

	TBARS	Carbonyl	Glucose	Creatine	Creatinine
Norharman	0.910*	0.849	-0.914*	-0.403	-0.083
Harman	0.951*	0.990**	-0.920*	0.050	0.229
CML	0.629	0.417	-0.509	0.115	0.444
CEL	0.992**	0.933*	-0.727	0.285	0.219

** $P<0.01$； * $P<0.05$；
